# Stable diesel microemulsion using diammonium ionic liquids and their effects on fuel properties, particle size characteristics and combustion calculations

**DOI:** 10.1038/s41598-024-57955-6

**Published:** 2024-04-02

**Authors:** H. A. El Nagy, Mahmoud Abd El-Aziz Mohamed

**Affiliations:** 1 Chemistry Department, Faculty of Science, Suez Canal University, Ismailia, 41522 Egypt; 2 Abu Sultan Thermal Power Plant, East Delta Electricity Production Company, Ismailia, Egypt

**Keywords:** Microemulsion fuel, Emulsifier, Ionic liquids, Waste cooking oil, Diquaternary ammonium, particle size, Ethanol diesel blends, Combustion calculations, Chemistry, Energy, Green chemistry, Organic chemistry, Chemical synthesis

## Abstract

Ecofriendly and stable Fuel Microemulsions based on renewable components were prepared through solubilizing ethanol in diesel and waste cooking oil blend (4:1). New diquaternary ammonium ionic liquids (3a & 3b) were synthesized through a quaternization reaction of the synthesized dihaloester with diethyl ethanolamine tridecantrioate and triethyl amine tridecantrioate, respectively. The chemical structures were elucidated by NMR spectroscopy. It was observed from DLS analyses that the ethanol particles in all samples have sizes between 4.77 to 11.22 nm. The distribution becomes narrower with the decrease in the ionic liquid concentrations. The fuel properties fall within the ASTM D975 acceptable specifications and are close to the neat diesel properties. The Cetane index were 53 and 53.5, heating values were 38.5 and 38.5 MJ/kg, viscosities were 2.91 and 2.98 mm^2^/s, densities were 8.26 and 8.29 g/mL and flash points were 49 °C and 48 °C for 3a1 and 3b1 microemulsions, respectively. The particle sizes of samples were examined by DLS for 160 days and they were significantly stable. The amount of ethanol solubilized increases with the increase in the amount of the synthesized ionic liquids and cosurfactant. The combustion calculations pointed out that the microemulsions 3a1 and 3b1 need 13.07 kg air/kg fuel and 12.79 kg air/kg fuel, respectively, which are less than the air required to combust the pure diesel. According to theoretical combustion, using ionic liquids saves the air consumption required for combustion and reduces the quantities of combustion products. The prepared microemulsions were successfully used as a diesel substitute due to their improved combustion properties than pure diesel and low pollution levels.

## Introduction

Diesel fuels have become widely used in many industries, including transportation and agricultural applications due to their high thermal efficiency^1^. Even though the diesel engine has high thermal efficiency, it emits a variety of pollutants into the atmosphere, including nitrogen oxides (NOx), sulphur (SOx), carbon oxides, particulate matter (PM), unburned hydrocarbons (HC)^2,3^.

As a result, numerous researchers are working to solve these issues and lessen the harmful effects of these systems using various tactics such as the addition of alcohols such as ethanol^4^ or the addition of nanoparticles to diesel fuel. Yusuf et al.^5^ examined the effects of blends of biodiesel and CeO_2_ nanoparticles on organic compounds and gaseous emissions from diesel engines. Biofuels are one sort of alternative fuel that is gaining popularity as a way to assist ensure future energy security while also reducing greenhouse gas emissions^6,7^. Apart from energy generated from municipal waste, another possible energy source is biofuel made from vegetable oil, which is currently available in significant quantities^8^.

The conversion of edible oil (such as soybean oil, canola oil, and palm oil) into biodiesel is the most common method of converting biofuel into automotive fuel. However, large-scale biodiesel production from edible oils may cause a global food supply imbalance, resulting in ecosystem devastation and economic and social hardships**.** As a result, researchers are interested in using waste cooking oil (WCO) as an alternative source of fuel for avoiding competition with feed crops^9^. El‑Sheekh et al.^10^ investigated the effect of adding bioethanol at different ratios to WCO biodiesel/diesel blends on solubility and stability.

Transesterification, pyrolysis, and microemulsion blending are some of the common methods for modifying the characteristics of suitable vegetable oil to make it better suited to diesel engines^11,12^. Transesterification, in general, yields unpurified by-products such as alcohol, glycerol, tri-, di-, and mono-glycerides, catalysts, and a substantial amount of polluted wastewater. As a result, biofuel sustainability production as a substitute for fossil fuels has been challenged^13^.

Fuel microemulsions have been proposed and studied as an alternate approach to generate fuels with adequate qualities while avoiding waste disposal issues. It is a simple technology with minimal energy consumption, is easy to operate, and gives 100% product^14–16^. It also can be utilized in the diesel engine directly without the need for any modifications in the engine system^17^. Furthermore, the emulsification process has been used to overcome the viscosity issues of vegetable oils^18^. Microemulsions are isotropic transparent and thermodynamically stable solutions^19^. Their thermodynamic stability distinguishes them from ordinary emulsions, as well as their substantially lower structural size (3–40 nm)^20^. Generally, the four main components of microemulsions are the oil phase, aqueous phase, surfactant, and cosurfactant^21^. Surfactants are used as an emulsifier in this process to prevent phase separation between the three components (WCO, diesel, and ethanol)^8^.

Surfactant selection for microemulsion fuels is one of the most difficult topics in this industry, as it has a significant impact on the pricing and specifications of the fuel produced. The chemical structure and the amount of the surfactant are two of the most important factors influencing the aqueous phase characteristics in diesel fuel microemulsions^17^. El‑Sheekh et al.^22^ used tri-n-butyl phosphate as a surfactant for improving the solubility and stability of ethanol/diesel blends for direct injection in diesel engine.

In recent years, using ionic liquids (ILs) as surfactants have been studied, as their properties may be modified by changing their cation or anion type^23,24^. ILs are low-temperature molten salts that are fully constituted of anions and cations near room temperature^25^. Wilkes and Zaworotko^26^ produced 1-ethyl-3-methylimidazolium tetrafluoroborates in the 1990s, and it was stable in both air and water. They also prepared a more hydrophobic 1-ethyl-3-methylimidazolium hexafluorophosphate at the same time. Following that, other types of ILs, such as azoles, pyridines^27^, and quaternary ammonium salts, were continually^28^. Low melting point, strong electrical conductivity, great stability, a large electrochemical conductivity, and wide liquid range distinguish ILs, making them useful in a variety of sectors and allowing them to replace some harmful organic compounds. Moreover, ILs are included in surfactant-free microemulsion systems because of their numerous benefits. Xu et al.^29^ investigated the system (BmimPF_6_/DMF/H_2_O), using the IL (BmimPF_6_) as the non-polar phase.

ILs are also known as green solvents since they are eco-friendly agents and more stable than surfactants and other chemical compounds^30^. Aside from the use of ILs as solvents in catalysis^31^ or synthesis^32^, the ability of amphiphilic structures association to form microemulsions, micelles, and liquid-crystalline phases has recently been studied^33^. Surfactant ILs based on pyridinium and imidazolium have been examined for their effect on the interfacial tension of the water/crude oil system^30,34^. These ILs substances were found to be useful in lowering the interfacial tension between crude oil and its solutions^35^.

ILs can be utilized as polar or non-polar solvents to produce stable fuel microemulsions, dependent on their anion and cation characteristics^23^. Zhang et al. studied the dispersion of [Bmim][BF4] ionic liquid as polar phase in P-xylene using nonionic surfactant^36^. Zech et al. studied a microemulsion containing ethyl ammonium nitrate and 1-butyl-3-methylimidazolium tetrafluoroborate as the polar phase in dodecane as a nonpolar phase^37^. Because of their exceptional solvent characteristics, using ILs are desirable as dispersed or continuous phases in microemulsions. Furthermore, in order to prepare microemulsions, ILs have been used as emulsifiers. ILs with long hydrocarbon chain work as amphiphilic compounds, making them excellent for developing the targeted microemulsion fuel^38^. Ionic liquid, 1-hexadecyl-3- methyl imidazolium bromide was used as a surfactant for enhanced oil recovery^39^. Limited research studies used ILs to enhance diesel microemulsion and its properities for usage as an alternate fuel.

In terms of thermal stability, volatility, and maintainability of physical and chemical properties, dicationic ILs have several benefits over conventional monocationic ILs^40^. They, therefore, offer good potential for application as high-temperature solvents, lubricants, stationary phases for gas chromatography, separation media, and catalysts for transesterification reactions^41,42^. When compared to their traditional cationic and anionic surfactant equivalents, it was found that these ILs had improved surface activity. It was because ILs' amphiphilic properties had improved in response to different alkyl chain lengths of the cationic and anionic groups^43^.

The main purpose of this study is to prepare new eco-friendly microemulsion fuels through dispersing ethanol in diesel/WCO with high stability using novel diquaternary ammonium ionic liquids as emulsifiers. The chemical structures of the synthesized ionic liquids are characterized by NMR spectroscopy. Dynamic light scattering (DLS) examinations of the emulsified ethanol particles provided information on their particle size and size distribution, confirming the stability of the prepared microemulsion fuels. Ternary phase diagram of the prepared microemulsions is also displayed. In order to ensure the stability of the prepared samples, particle sizes were examined after storage for 160 days. Additionally, effect of the amount of the synthesized ionic liquids and cosurfactant on ethanol solubilization was investigated. The fuel properties of microemulsion fuel samples were investigated and compared with the properties of ASTM limits and the neat diesel fuel. The combustion calculations were performed to calculate the chemical energy content and the emissions from the microemulsion fuel.

## Experimental

### Materials

Polyethylene glycol 400 and chloroacetic acid were bought from Merck. P-toluene sulfonic acid, triethanolamine and Myristic acid were purchased from Sigma Al-Drich Co. The solvents utilized in synthesis were laboratory grade with purity > 99% and involved toluene, and benzene. The utilized diesel was acquired from a local gas station and the household waste cooking oil (WCO) was collected. The co-surfactant used was 1-butanol (purity 99%) was utilized without any extra purification.

### Ionic liquids synthesis procedure

#### Synthesis of dichloroethandioate polyethylene glycol (**1**)

Polyethylene glycol 400 (0.01 mol) and chloroacetic acid (0.022 mol) were refluxed at 110 °C in the presence of toluene as a solvent and 0.001 wt% P-toluene sulfonic acid. The reaction was finished when the theoretical amount of water was removed. The solvent was vacuum evaporated to obtain a dark brown liquid, product **(1).**

#### Synthesis of the diethyl ethanolamine tridecantrioate (**2a**) and triethyl amine tridecantrioate (**2b**)

Triethanolamine (0.01 mol) was reacted with myristic acid (0.02 mol) and (0.03 mol) to obtain the triethanolamine diester (2a) and triester (2b), respectively. The mixture was refluxed at 110 °C in the presence of toluene as solvent and 0.001 wt % P-toluene sulfonic acid. The reaction was completed when the theoretical amount of water was removed. The mixture was cooled and toluene was vacuum evaporated to obtain the diethyl ethanolamine tridecantrioate (2a) and triethyl amine tridecantrioate (2b).

#### Synthesis of the diquaternary ammonium branched ionic liquid (**3a**) and (**3b**)

The prepared dihaloester (**1**) (0.01 mol) was refluxed with 0.022 mol of **2a** and **3a** individually in benzene as a quaternizing agent. Two drops of pipridine were added as a catalyst. The mixture was refluxed for 24 h at 80 °C under stirring. Then, the obtained products were filtered off, recrystallized from benzene and dried to yield the dicationic ammonium ionic liquids products (**3a** & **3b)** with a yield of 88 and 90%, respectively.

### Spectroscopic chemical structure characterization

^13^C-NMR and ^1^H-NMR spectra Analyses were performed on a 400 MHz spectrometer, DRX-400 Bruker Advance, using the DMSO-d6 as a solvent for elucidating the chemically expected structures of the synthesized dicationic ammonium ionic liquids (3a & 3b).

### Preparation of microemulsion fuels

WCO, diesel, and ethanol were combined to make the produced microemulsion fuels in the presence of the prepared ionic liquids (3a and 3b) which served as emulsifiers. The mixture of diesel/WCO (4:1) was utilized as an oil phase. The ionic liquids (3a and 3b) were dissolved in ethanol at concentrations of 2500 ppm (labeled as 3a1 & 3b1 microemulsion samples), 5000 ppm (3a2 & 3b2 samples), 7500 ppm (3a3 & 3b3 samples) and 10,000 ppm (3a4 & 3b4 samples). The co-surfactant (1-butanol) was used to create a clear appearance of the system. Mixing these components was performed at room temperature, stirring at 850 rpm for 10 min.

### The dynamic light scattering (DLS) analyses

The produced microemulsion samples were subjected to photon correlation spectroscopy using Dynamic Light Scattering (DLS) (particle size analyzer, Nano ZN, Zetasizer device, Malvern P analytical Ltd, UK) at 25 °C at a fixed angle of 173° to determine their particle size and size distribution. The prepared microemulsions were examined for three separate tests.

### Fuel properties of microemulsions

For samples of diesel, diesel/WCO mixture, 3a1, and 3b1 microemulsion fuels, physical–chemical properties were tested. Using the Tamson Tv4000MkIL and the Densitometer A. Kruss Optronic standards of ASTM D445 and D5002, respectively, the kinematic viscosity and density were calculated. According to the ASTM D4294 standard, Horiba SLFA-6800, sulfur content was measured and the ASTM D4006 standard, Dean Stark Stanhope-Seta, is used for measuring water content. The ASTM D2500 is used for testing the cloud point. According to ASTM D4809 and ASTM D93 standards, Auto flash point Stanhope-seta 35000-04, respectively, the heating values and flash points were calculated. Moreover, the ASTM D976 is used for calculating the cetane index.

## Results and discussions

**Figure 1:**
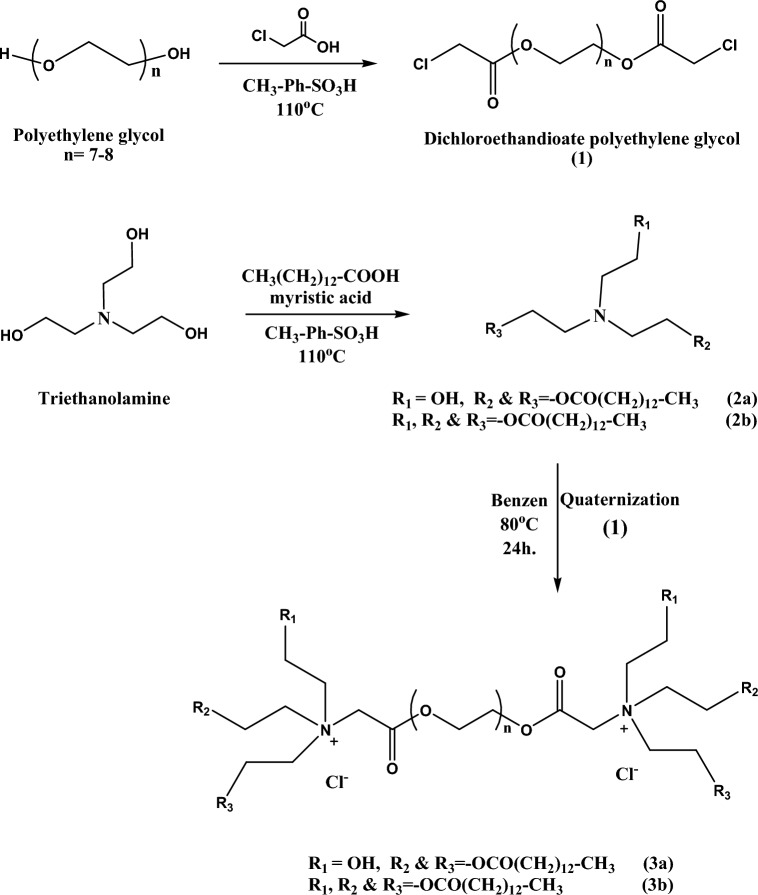
Synthesis of the branched diquaternary ammonium ionic liquids (3a & 3b).

Two new eco-friendly diquaternary ammonium ionic liquids (3a and 3b) based on polyethylene glycol were synthesized through quaternization reaction of dihaloester (1) with diethyl ethanolamine tridecantrioate (2a) and triethyl amine tridecantrioate (2b), respectively. Ethanol was emulsified with a diesel/WCO mixture using the produced ionic liquids to prepare environmentally friendly fuel microemulsions. Figure 1 represents the ionic liquids suggested chemical structure.

### Spectroscopic characterization of the prepared ionic liquids

#### Spectroscopic characterization of dichloroethanoate polyethylene glycol (**1**)

**Figure 2:**
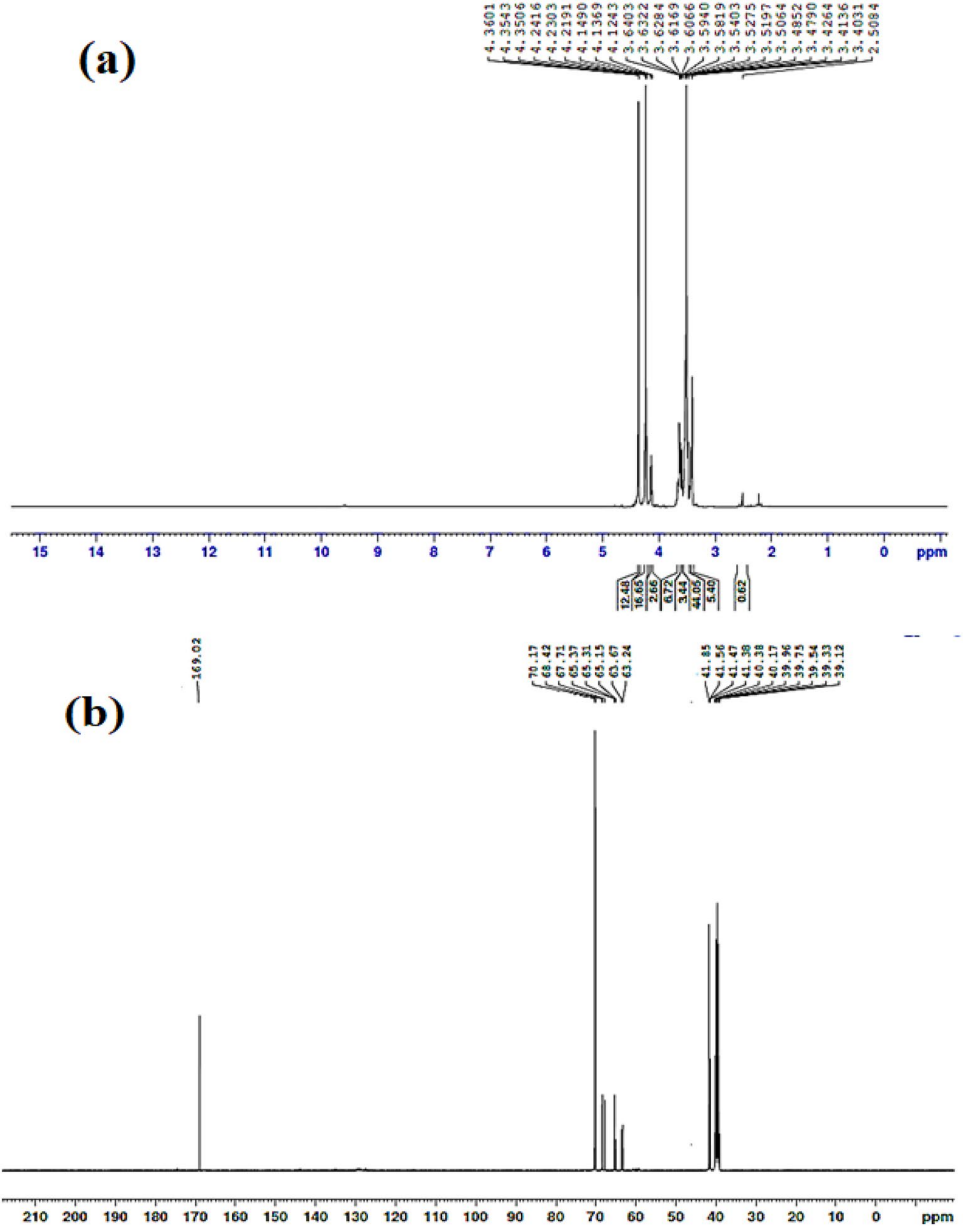
Chemical structure spectroscopic characterization of Dichloroethanoate polyethylene glycol (1): (**a**) ^1^H-NMR and (**b**) ^13^C-NMR.

Figure 2a represents the ^**1**^**H-NMR, 400 MHZ, DMSO-d**_**6**_ at chemical shifts of 4.12–4.14 ppm (s, Cl-CH_2_-CO), 4.21–4.36 ppm (ethoxy hydrogens, O-CH_2_-CH_2_). Figure 2b represents the ^**13**^**C-NMR, DMSO-d**_**6**_**, 400 MHZ** at signals 41.38–41.85 ppm (CH_2_-Cl), 63.24–70.17 ppm (O-CH_2_-CH_2_-O) and 169.02 ppm (CO-CH_2_-Cl).

#### Spectroscopic characterization of the triethanolamine diester (**2a**) and triester (**2b**)

The ^**1**^**H-NMR, 400 MHZ, DMSO-d**_**6**_ of **(2a)** is at chemical shifts of 0.86 ppm (CH_3_-(CH_2_)_10_), 1.24 ppm (CH_3_-(CH_2_)_10_), 1.49–1.50 ppm (O-CO-CH_2_-CH_2_), 2.09 ppm (–OH), 2.27 ppm (O-CO-CH_2_-CH_2_), 2.38 ppm (N-CH_2_-CH_2_–), 3.54 ppm (HO-CH_2_) and 4.05–4.12 ppm (CH_2_-O). The ^**13**^**C-NMR, DMSO-d**_**6**_**, 400 MHZ** of **(2a)** is at signals of 14.39 ppm (CO-CH_2_-CH_n_-CH_2_-CH_3_), 22.56 ppm (CO-CH_2_-CH_n_-CH_2_-CH_3_), 29.18 ppm (CO-CH_2_-CH_n_), 33.97 ppm (CO-CH_2_), 63.67 ppm (N-CH_2_-CH_2_-O), 68.35 ppm (N-CH_2_-CH_2_-O), 60.01 ppm (N-CH_2_-CH_2_-OH), 172.93 ppm (O-CO).

The ^**1**^**H-NMR, 400 MHZ, DMSO-d**_**6**_ of **(2b)** is at chemical shifts of 0.86 ppm (CH_3_-(CH_2_)_10_), 1.24 ppm (CH_3_-(CH_2_)_10_), 1.48–1.50 ppm (O-CO-CH_2_-CH_2_), 2.29 ppm (O-CO-CH_2_-CH_2_), 2.40 ppm (N-CH_2_-CH_2_-) and 4.05–4.12 ppm (CH_2_-O). The ^**13**^**C-NMR, DMSO-d**_**6**_**, 400 MHZ** of **(2b)** is at signals of 14.37 ppm (CO-CH_2_-CH_n_-CH_2_-CH_3_), 22.57 ppm (CO-CH_2_-CH_n_-CH_2_-CH_3_), 29.50 ppm (CO-CH_2_-CH_n_), 34.15 ppm (CO-CH_2_), 63.67 ppm (N-CH_2_-CH_2_-O), 68.52 ppm (N-CH_2_-CH_2_-O), 174.87 ppm (O-CO).

#### Spectroscopic characterization of the diquaternary ammonium ionic liquid (**3a**) and (**3b**)

**Figure 3:**
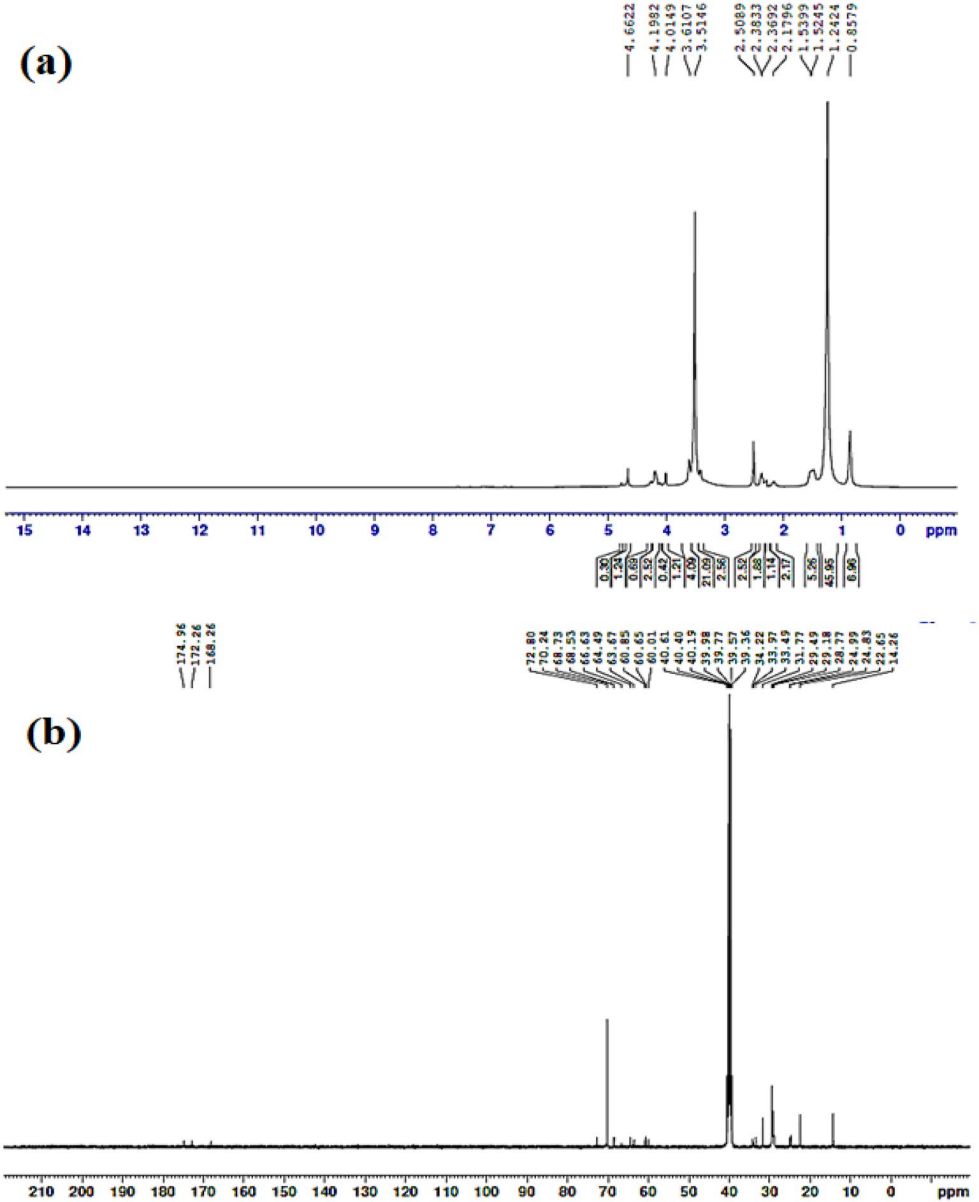
Chemical structure spectroscopic characterization of Diquaternary ammonium ionic liquid **(3a):** (**a**) ^1^H-NMR and (**b**) ^13^C-NMR.

Figure 3a represents the ^**1**^**H-NMR, 400 MHZ, DMSO-d**_**6**_ of **(3a)** at chemical shifts of 0.85 ppm (CH_3_-(CH_2_)_10_), 1.24 ppm (CH_3_-(CH_2_)_10_), 1.52–1.53 ppm (O-CO-CH_2_-CH_2_), 2.17 ppm (^1^OH), 2.36–2.38 ppm (N^+^-CH_2_-CH_2_-O), 3.61 ppm (HO-CH_2_), 4.01 ppm (CO-CH_2_-N^+1^), 4.19 ppm (N^+^-CH_2_-CH_2_-O) and 4.66 ppm (O-CH_2_-CH_2_-O). Figure 3b represent the ^**13**^**C-NMR, DMSO-d**_**6**_**, 400 MHZ of (3a)** at signals of 14.26 ppm (CO-CH_2_-CH_n_-CH_2_-CH_3_), 22.65 ppm (CO-CH_2_-CH_n_-CH_2_-CH_3_), 28.77–29.49 ppm (CO-CH_2_-CH_n_), 33.49–34.22 ppm (CO-CH_2_), 60.01 ppm (N^+^-CH_2_-CH_2_-OH), 60.65–60.85 ppm (CO-CH_2_-N^+^), 64.49 ppm (N^+^-CH_2_-CH_2_-O), 68.53–68.73 ppm (N^+^-CH_2_-CH_2_-O), 70.24–72.80 ppm (O-CH_2_-CH_2_-O) and 172.26–174.96 ppm (O-CO).

**Figure 4:**
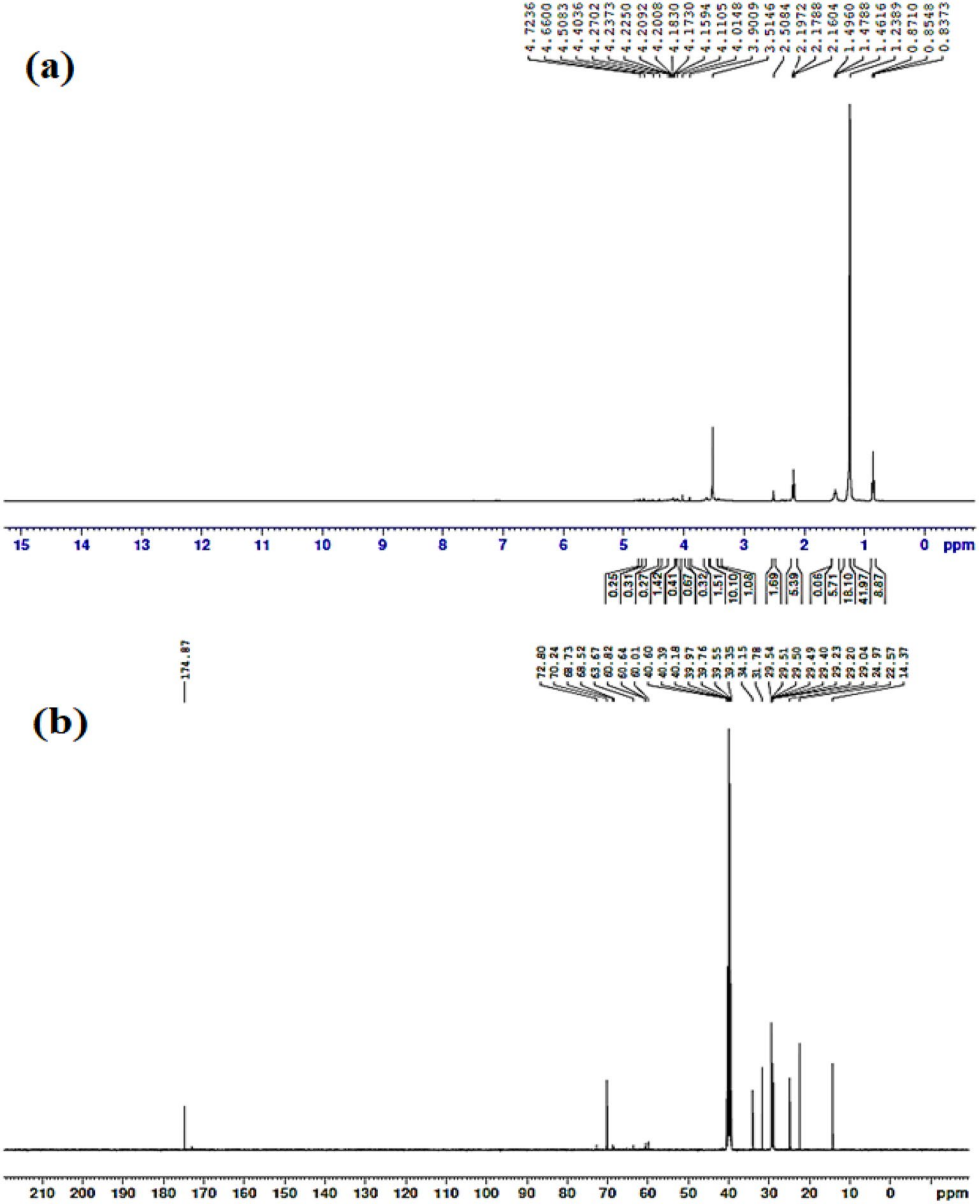
Chemical structure spectroscopic characterization of Diquaternary ammonium ionic liquid **(3b):** (**a**) ^1^H-NMR and (**b**) ^13^C-NMR.

Figure 4a represents the ^**1**^**H-NMR, 400 MHZ, DMSO-d**_**6**_ of **(3b)** at chemical shifts of 0.83–0.87 ppm (CH_3_-(CH_2_)_10_), 1.23 ppm (CH_3_-(CH_2_)_10_), 1.46–1.49 ppm (O-CO-CH_2_-CH_2_), 2.16–2.19 ppm (N^+^-CH_2_-CH_2_-O), 4.01 ppm (CO-CH_2_-N^+^-), 4.20 ppm (N^+^-CH_2_-CH_2_-O), 4.66 ppm (O-CH_2_-CH_2_-O). Figure 4b represents the ^**13**^**C-NMR, DMSO-d**_**6**_**, 400 MHZ of (3b)** at signals of 14.37 ppm (CO-CH_2_-CH_n_-CH_2_-CH_3_), 22.57 ppm (CO-CH_2_-CH_n_-CH_2_-CH_3_), 29.04–29.54 ppm (CO-CH_2_-CH_n_), 31.78–34.15 ppm (CO-CH_2_), 60.82 ppm (CO-CH_2_-N^+^), 63.67 ppm (N^+^-CH_2_-CH_2_-O), 68.52–68.73 ppm (N^+^-CH_2_-CH_2_-O), 70.24–72.80 ppm (O-CH_2_-CH_2_-O) and 174.87 ppm (O-CO).

### Ternary phase diagram and solubilization of ethanol

**Figure 5:**
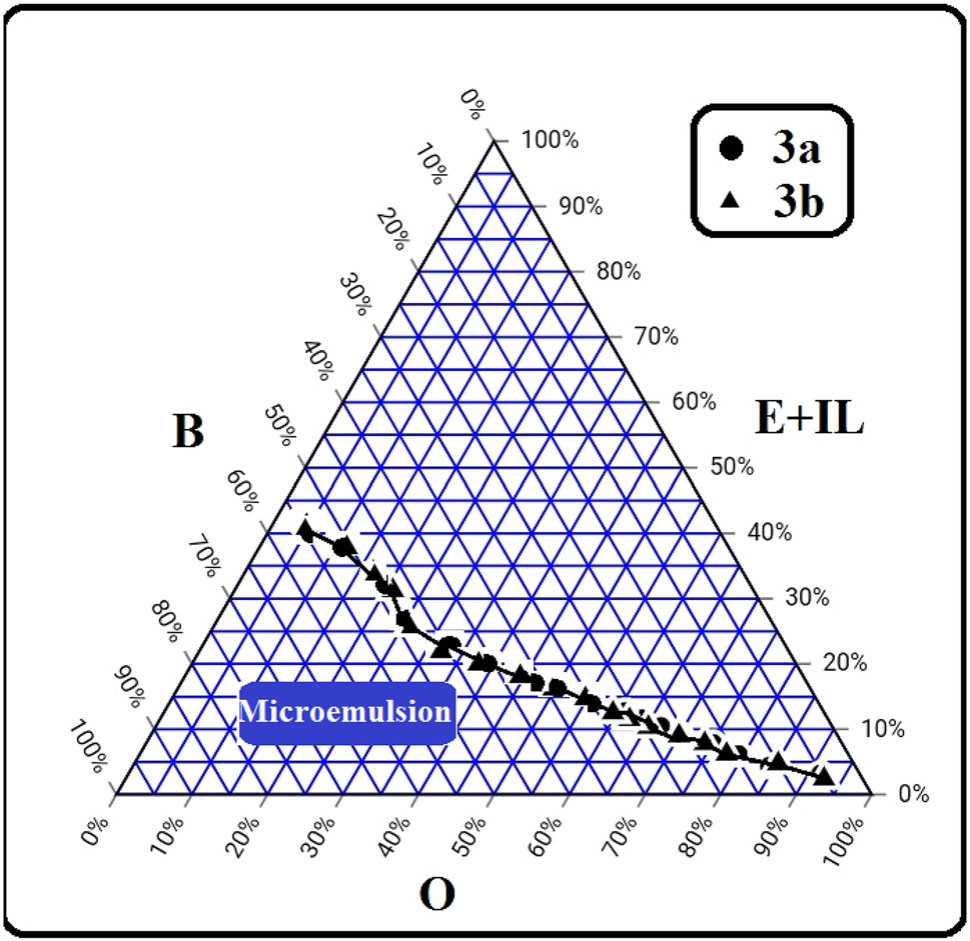
Ternary phase diagram of diesel/WCO (O), ethanol & synthesized ionic liquids (E + IL) and 1-butanol (**B**) at room temperature.

The triangle diagram showed at Fig. 5 was used to detect the phase change of microemulsion samples and to determine the microemulsion area after addition of different proportions of ethanol & IL (E + IL), diesel/WCO (O) and butanol (B). It was observed that the synthesized 3a and 3b have the same behavior as emulsifiers. Moreover, the microemulsion area decreases as the amount of diesel/WCO (O) increases at the right side of the ternary diagram. The microemulsion area represent nearly 34.5% of the phase diagram. In this study, the microemulsion which have components O = 77%, E + IL = 4% and B = 19% was selected to study the effect of the synthesized ionic liquids as emulsifiers.

**Figure 6:**
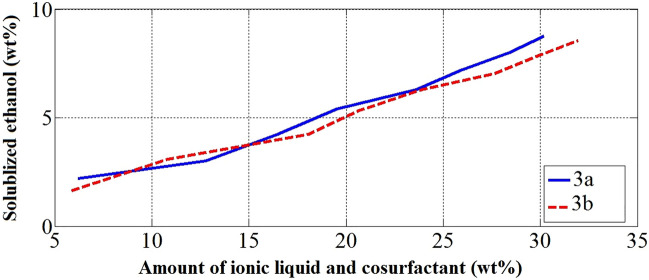
Effect of the synthesized ionic liquids and cosurfactant amounts on ethanol solubilization.

The synthesized ionic liquids (3a & 3b) have almost close effect on solubilizing ethanol within diesel/WCO mixture with the help of the cosurfactant as presented in Fig. 6. The amount of ethanol solubilized increases with the increase in the amount of ionic liquid and cosurfactant. The effect of ionic liquids on solubilization is very close at low percentages (< 15 wt%) of the amount of ionic liquids and cosurfactant, while at higher percentages, there is a slight divergence between the two curves. As a result, at the same percentage of ethanol solubilized within diesel/WCO, the mixture needs to higher amount of 3b and cosurfactant compared to the amount of 3a and cosurfactant.

### Dynamic light scattering (DLS)

The particle size of the dispersed phase is well recognized to be one of the major determinants of the stability of ethanol in diesel microemulsion particles. The size and distribution of the ethanol particles in the prepared fuel microemulsions were evaluated using dynamic light scattering. The homogenies of prepared microemulsions depend on the size of ethanol particles and the variations in sizes. The better homogeneity is achieved at minimum particle size and when the variation in these particles is limited.

**Figure 7:**
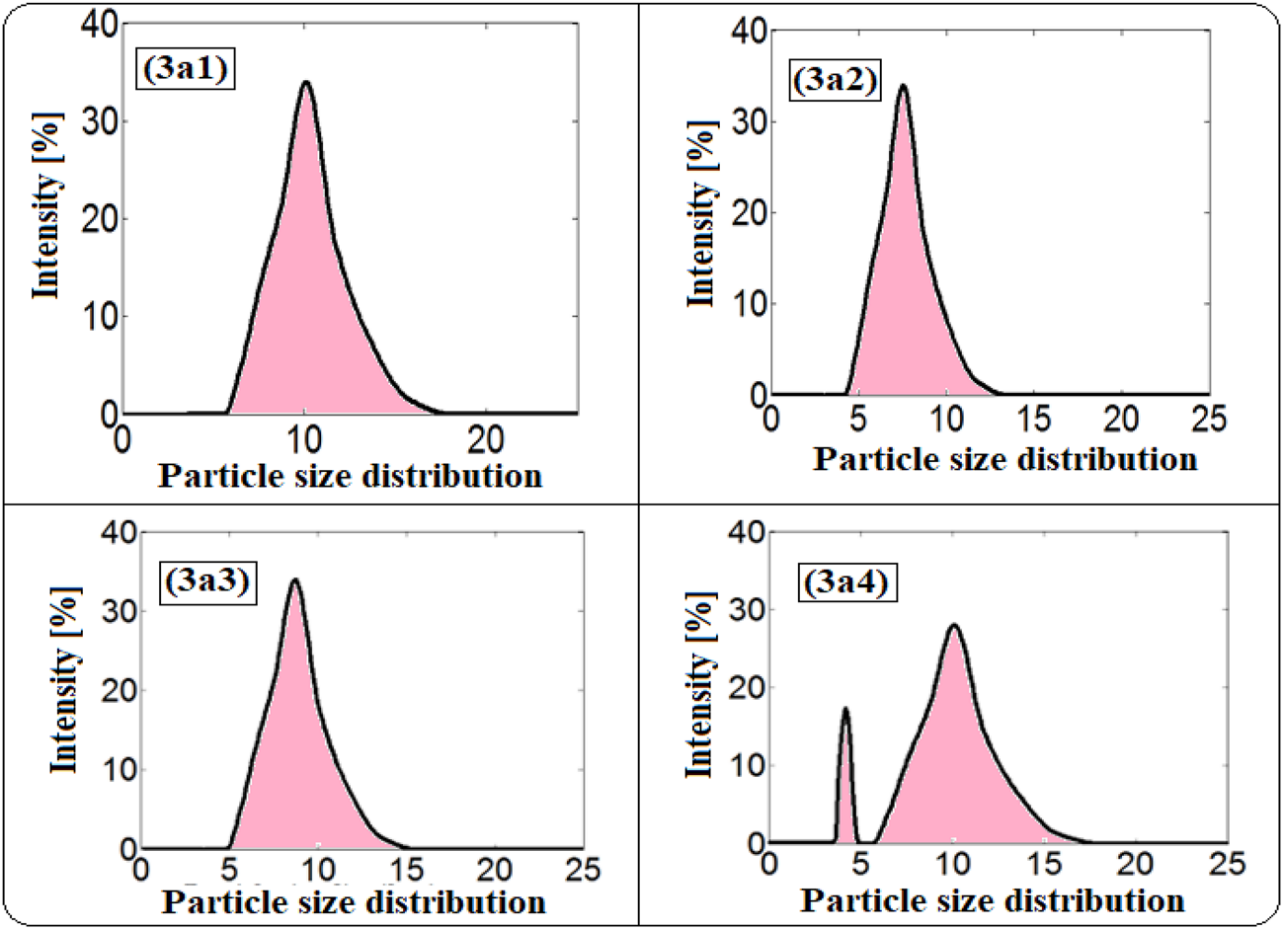
DLS curves of the size distribution for microemulsion samples using 3a ionic liquid as an emulsifier.

**Figure 8:**
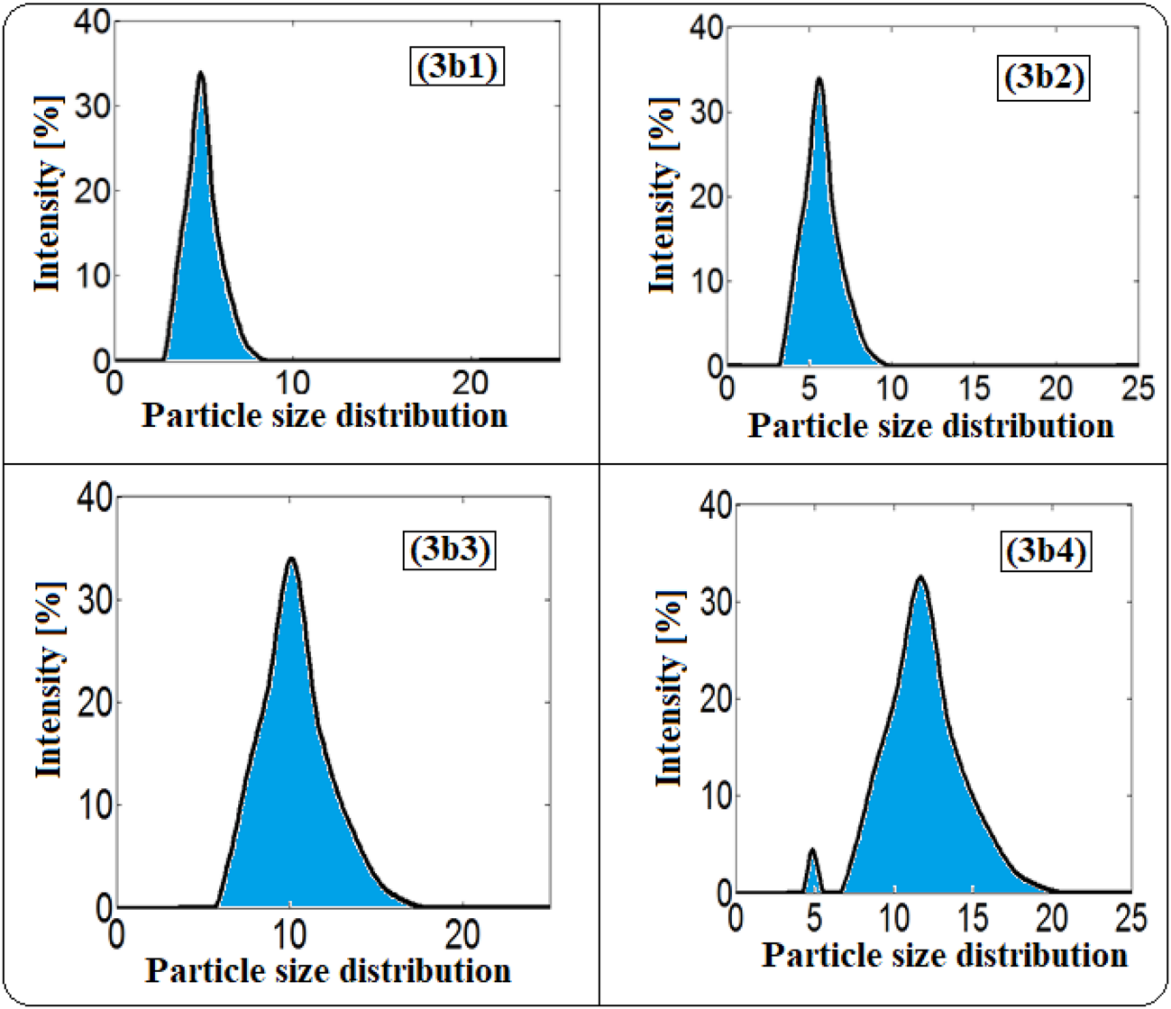
DLS curves of the size distribution for microemulsion samples using 3b ionic liquid as an emulsifier.

The diameter size of ethanol particles diffused in the diesel phase and their size distribution was displayed in Figs. 7 and 8 and Tables 1 and 2. Rosen^44^ defined emulsions with droplet sizes less than 200 nm as microemulsions, while Tadros^45^ classified them as less than 50 nm. It was observed that the ethanol particles in all samples have sizes between 4.77 to 11.22 nm. The size of ethanol particles increases with increasing the concentration of ethanol and ionic liquids (3a & 3b). It is well known that dicationic ILs have more benefits over conventional monocationic ILs and the ILs with long hydrocarbon chains work as good emulsifiers for developing stable microemulsion fuel^46,47^.

**Table 1:** The mean particle sizes, size distribution, and statistical values for 3a microemulsion samples.

Sample	Min (nm)	Max (nm)	The range (nm)	The mean (nm)	The variance	The skew	The kurtosis
3a1	3.12	11.70	8.58	6.40	1.55	1.55	0.94
3a2	3.62	13.55	9.93	7.41	2.08	0.49	3.16
3a3	4.19	15.67	11.48	8.58	2.79	0.49	3.16
3a4	4.19	18.17	13.98	8.94	7.84	− 0.25	2.54

**Table 2:** The mean particle sizes, size distribution and statistical values for 3b microemulsion samples.

Sample	Min (nm)	Max (nm)	The range (nm)	The mean (nm)	The variance	The skew	The kurtosis
3b1	2.33	8.72	6.39	4.77	0.86	0.49	3.16
3b2	2.70	10.10	7.40	5.52	1.16	0.49	3.16
3b3	3.62	21.04	17.42	9.94	3.74	0.48	3.16
3b4	4.19	18.17	13.98	11.22	6.67	− 0.11	3.54

In addition, the 3b1 sample showed the lowest size of ethanol particles (4.77 nm). Additionally, it was observed that samples 3b1 and 3b2 (containing low concentrations of ethanol and IL) have lower particle sizes than the corresponding samples 3a1 and 3a2 with 25%. However, there is an increase in the particle sizes of samples 3b3 and 3b4 (containing high concentrations of ethanol and IL) than the corresponding samples 3a3 and 3a4 by 20%. Moreover, in the case of 3a microemulsion samples, the particle size increases gradually with increasing in the concentration of 3a ionic liquid from 3a1 to 3a4. Though, in the case of 3b microemulsion samples, the particle size increases gradually in 3b1 and 3b2 samples with increasing in the concentration of 3b ionic liquid but, the particle size was nearly doubled in 3b3 and 3b4 samples. This is because of the increased number of long alkyl chains in 3b compared with 3a ionic liquid which improves the 3b surface activity as an emulsifier. But, at high concentrations of 3b ionic liquids, the particle size increase owing to the crowding caused by the increased number of long alkyl chains. This indicates that the structures of the synthesized ionic liquids have a clear and good effect on the particle size and microemulsification stability.

**Figure 9:**
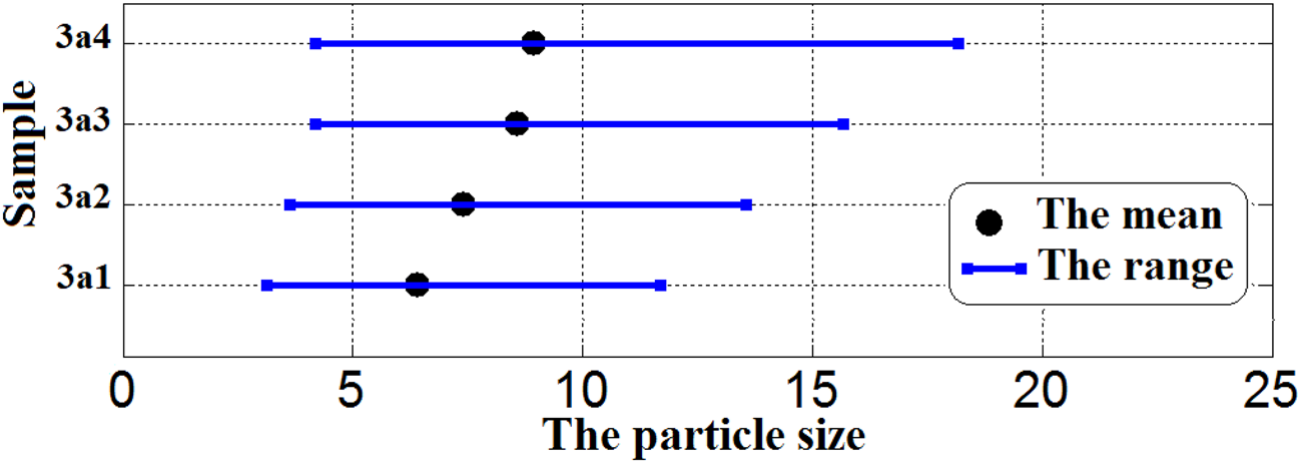
The variation of size distribution characteristics with the mean particle size among the 3a microemulsion samples.

**Figure 10:**
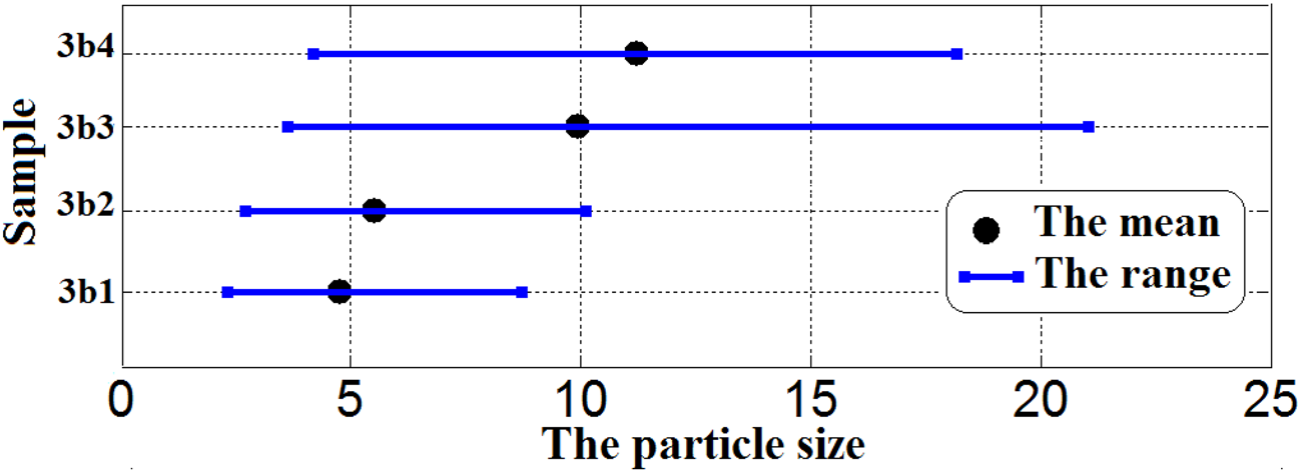
The variation of size distribution characteristics with the mean particle size among the 3b microemulsion samples.

Figures 9 and 10 illustrate the variation of the size distribution characteristics among the microemulsion samples. The percentage of ionic liquid affected the mean particle size and its distribution. The distribution becomes narrower with the decrease in the concentration of ionic liquids. The two synthesized ionic liquids show the same behavior.

### Stability of the prepared fuel microemulsions

**Figure 11:**
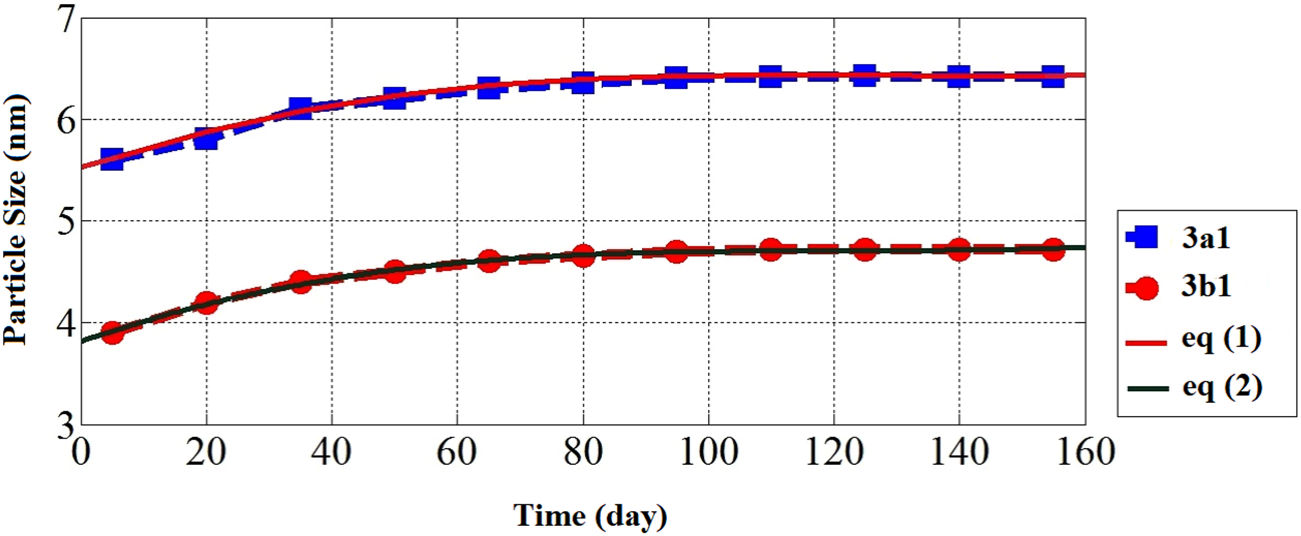
The particle sizes with time for the prepared microemulsions.

The stability of microemulsion fuel is a very important factor in its storage. The prepared microemulsion samples showed no phase separation for more than a year. In addition, their particle sizes were examined every 15 days and it was observed that they were significantly stable as shown in Fig. 11. In the first five days, the mean particle sizes were 5.5 and 3.8 nm for 3a1 and 3b1 microemulsion samples, respectively. After 15 days, the mean particle sizes became 5.8 and 4.2 nm for 3a1 and 3b1 microemulsion samples, respectively. After 80 days, it was observed that the mean particle sizes gradually increase to 6.4 and 4.6 nm for 3a1 and 3b1 microemulsion samples, respectively, and remained nearly stable at these values of particle sizes for 160 days, proving the suitability of adding the synthesized ionic liquids to enhance the solubilizing of ethanol in diesel fuel and to enhance the microemulsion's physical stability. In addition, a fitting curve was established from the experimental results to predict the extent of the increase in particle size over a period of more than 160 days. The Eqs. (1) and (2) represent the curve fitting to predict the particle size (*P*) as a function of time by days (*X*) for 3a1 and 3b1 microemulsion samples, respectively.

1 $${P}_{3a1}=\left(4.3*{10}^{-7}\right){X}^{3}-\left(1.7*{10}^{-4}\right){X}^{2}+\left(2.2*{10}^{-2}\right){X}+5.5$$

2 $${P}_{3b1}=\left(3*{10}^{-7}\right){X}^{3}-\left(1.3*{10}^{-4}\right){X}^{2}+\left(1.7*{10}^{-2}\right){X}+3.9$$

where, *P* is the particle size (nm) and *X* is the time (days).

According to Eqs. (1) and (2), the change in particle size can be predicted within a year (365 day). The particle size is slightly increases to 11.8 nm and 7.4 nm for 3a1 and 3b1, respectively.

It was concluded that the ionic liquids' chemical structures have a role in the stability. The diquaternary ammonium cations and the ethylene oxide spacer in the synthesized ionic liquids interact strongly with ethanol particles and butanol. The stability of the microemulsion system is also influenced by the length and number of the hydrocarbon chains. Ionic liquids with longer and several hydrocarbon chains have the potential to be better emulsifiers and surface-active agents. As a result, the micelles that are created are more stable, which prevents the leakage of ethanol particles in the oil phase and prevents phase separation.

### Fuel properties of microemulsions

Physical–chemical characteristics for diesel, diesel/WCO blend, 3a1, and 3b1 microemulsion samples were investigated and compared with the ASTM D975 minimal standards as presented in Table 3.

**Table 3:** Fuel properties measurements for microemulsion samples.

Property	ASTM D975	Diesel	Diesel/WCO	3a1	3b1
Cetane index	40	55	54	53	53.5
Heating value (MJ/kg) (experimental)	–	41.10	40	38.5	38
Viscosity @ 40 °C (mm^2^/s)	1.9–4.1	2.17	6.00	2.91	2.98
Density@ 25 °C (g/mL)	–	8.343	8.633	8.262	8.293
Cloud point (^o^C)	–	− 3.3	− 4	− 3	− 2.8
Sulfur content (mg/kg)	50	43	35	30	31
Water and sediments (%v/v)	0.05	0.04	0.07	0.18	0.18
Flash point (^o^C)	52	56	70	49	48

The cetane number represents the fuel's ignition delay or period between the start of fuel injection and the start of combustion. The longer ignition delay would cause uncontrolled combustion and increase NOx emissions^48^. Microemulsions' cetane number is lower because alcohols have a lower cetane number^49^. It has been reported that addition of 17.4% aqueous ethanol in soyoil ethanol microemulsions reduces the cetane number from 37.9 to 29.8^50^.

In the present study, the cetane index (ASTM D976) is used in place of the cetane number. Although the cetane index of the microemulsion fuels was lower than those of diesel, they were still in the ASTM D975 acceptable minimum standards. The microemulsion fuels did not generate engine knock even though their lower cetane index indicated an ignition delay. The formed vapors were of alcohol which has low heating value than diesel, and this causes a lower peak temperature.

The heating value is a crucial property used to calculate the amount of energy released when it burns completely. The heating values of 3a1 and 3b1 samples vary between 38.5 MJ/kg to 38 MJ/kg, respectively, while the heating value of diesel is 41.10 MJ/kg. The addition of ethanol and butanol to the diesel/WCO mixture caused the heating values of 3a1 and 3b1 to somewhat decrease (7%). The results obtained by Chandra and Kumar^51^ displayed that the calorific value of plain diesel was 46.85 MJ/kg, whereas the ethanol-ethyl acetate-diesel microemulsions showed a reduction of 30.47–45.7 MJ/kg at different ratios of ethanol.

The results showed that the kinematic viscosity of diesel/WCO was the highest, while that of 3a1 and 3b1 samples were within the maximum ASTM D975 limit. The viscosity of the diesel/WCO mixture has significantly decreased as a result of the microemulsification of ethanol. Because ethanol has a lower viscosity than neat diesel, it is added to mixtures of vegetable oils/diesel to modify the viscosity. Canola and palm oil-based microemulsions containing 50% (v/v) of the corresponding vegetable oils need 24% (v/v) of ethanol to decrease their viscosity^52^. The 3a1 and 3b1 microemulsion fuels have kinematic viscosities that are slightly higher than neat diesel fuel, although their values still fall within the ASTM D975 acceptable specifications. The 3a1 and 3b1 microemulsion samples were observed to have a density that is close to neat diesel with a decrease of 0.97%. The atomization, the distribution of the droplet sizes, and ultimately the evaporation of the fuel in an engine would be influenced by the density and kinematic viscosity values of the fuel. Thus, these properties should have values comparable to the neat diesel.

**Figure 12:**
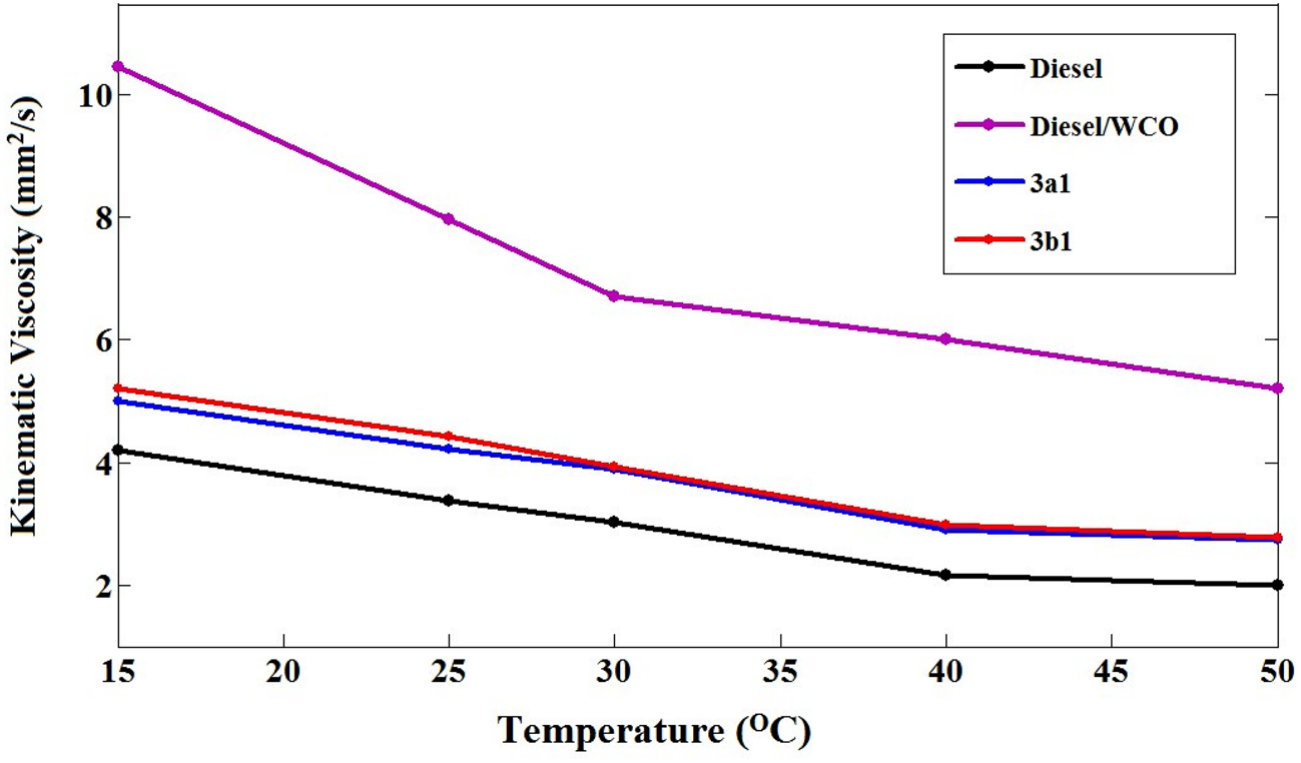
The kinematic viscosity of the prepared samples at various temperatures.

At various temperatures, the viscosity of the prepared samples was tested as shown in Fig. 12. The viscosity of the prepared samples reduces as the temperature increases. Moreover, no noticeable variation in viscosities has been found for 3a1 and 3b1 samples, but the viscosity values of 3b1 are slightly higher than that of 3a1 due to the more hydrophobic chains in 3b emulsifier.

Because the cloud points of the microemulsions were comparable to those of neat diesel, they could be working without any problems with cold starts. The addition of alcohol to the microemulsions also resulted in a small decrease in the flash points. It would therefore require careful storage and treatment. The water content values demonstrate that the neat diesel fuel and the diesel/WCO mixture had lower water contents than the 3a1 and 3b1 microemulsion fuels. This suggests that the addition of ethanol and butanol to the microemulsion fuels' compositions is the primary cause of the increase in water content values.

Due to the burning of fuel, sulfur content contributes to environmental pollution. The sulfur content results demonstrate that, in comparison to neat diesel fuel and a diesel/WCO blend, the sulphur levels of the 3a1 and 3b1 microemulsion fuels were lower. Microemulsion fuels are therefore regarded as green fuels.

### Theoretical calculations of microemulsion combustion stoichiometry

The purpose of these calculations is to calculate the chemical energy content and the emissions from the microemulsion fuel. The energy released by the theoretical oxidation of the fuel is estimated by the following calculations for 3a1 & 3b1 microemulsion samples. The effect of adding ionic liquids as emulsifiers on the combustion of diesel was indicated in Tables 4, 5, 6.

**Table 4:** Chemical composition of fuel.

Fuel	C [%]	H [%]	O [%]	H_2_O [%]	N [%]	CL [%]	S [%]	Ash [%]
Diesel	85.25	11.25	–	0.25	–	–	1.75	1.5
WCO	77.91	11.69	10.36	–	0.04	–	–	–
IL (3a)	63.08	10.39	20.53	–	1.73	4.27	–	–
IL (3b)	66.54	10.8	17.9	–	1.36	3.4	–	–
Ethanol	52.2	13.1	34.7	–	–	–	–	–
Butanol	64.87	13.52	21.61	–	–	–	–	–

**Table 5:** Chemical composition of the prepared microemulsion fuel.

Microemulsion sample	C [%]	H [%]	O [%]	H_2_O [%]	N [%]	CL [%]	S [%]	Ash [%]
3a1	78.86	11.85	7.0	0.13	0.23	5.4 * 10^–5^	1.2	1.2
3b1	78.03	11.5	6.8	0.10	0.54	4.31 * 10^–5^	1.1	1.1

**Table 6:** Combustion calculations.

Fuel	kg air/ kg fuel	Total product/kgf	HHV (MJ/kgf)	LHV(MJ/kgf)
Diesel	14	4.21	45.6	43
3a1	13.07	4	42.84	39.2
3b1	12.79	3.917	41.85	39.3

Table 4 shows the proportions of the elements (carbon, hydrogen, oxygen, water, nitrogen, chlorine, sulfur, and the percentage of ash) that make up the diesel, WCO, ionic liquids, ethanol and cosurfactant.

Oxygen is the reactive element of air in combustion. In general, it is accurate to say that air is made up of 21% oxygen and 79% inert gases that are assumed to be nitrogen (also known as apparent or atmospheric nitrogen). There are 3.773 mol of atmospheric nitrogen for every mole of oxygen in the air^53^.

Given that air has a molecular weight of 28.96, which is typically estimated at 29, it contains both nitrogen and oxygen, both of which have molecular weights of 28 and 32, respectively. Nitrogen was present in the air, but when the products exist at low temperatures, the reaction has less of an impact on the nitrogen^53^. Take into account the complete combustion of the fuel proportions for microemulsion in Table 5.

When using a ratio of 4% of the mixture of ionic liquids (2500ppm) with ethanol, a proportion of 19% of butanol, and a ratio of 77% as a mixture of diesel and WCO as a mixing ratio of 4:1, it affects the proportions of the fuel components of carbon, hydrogen, oxygen, water, nitrogen, chlorine, sulfur and the ash percentage as shown in Table 4. It is noticed from Table 5, the percentage calculated of chlorine (permanently present in the prepared fuel) tends to be zero and it will be ignored during the calculations. In addition, assuming that the amount of heat generated during the burning does not generate nitrogen oxides. The elements included in the combustion equations are carbon, hydrogen, carbon, and oxygen.

These ratios have an impact on combustion calculations. Through theoretical calculations of pure diesel fuel and the use of fuel prepared from diesel, ionic liquids, ethanol, and butanol. The change in the proportions of the combustible elements of carbon, hydrogen and sulfur, and the proportions of oxygen were indicated in Table 5.

Theoretical air supply per kg of fuel [= (100/23)((8/3)C + 8H_2_ + S)-O_2_) kg] as shown in Table 6, the amount of air required to burn the prepared fuel for complete combustion is lower than the amount required in the case of using pure diesel fuel. We find that the amount of air required for burning 3a1 microemulsion fuel is less by 6.6% than the amount of air used to burn the pure diesel, while it reaches 8.6% lower than the pure diesel in the case of 3b1 microemulsion fuel.

Moreover, the amount of air required for combustion in the pure diesel and the microemulsion samples produce different amounts of combustion products. It was found that the combustion products decrease by approximately 4.98% when burning 3a1 and decrease by 6.88% in the case of 3b1.

Through combustion calculations, it was noticed that the higher and lower calorific value depends on the fuel composition. The lower the percentage of carbon, the lower the calorific value. It decreases by 7.53% and 8.77% in the case of 3a1 and 3b1, respectively, as compared with pure diesel.

3 $${\text{H}}.{\text{C}}.{\text{V}}. \, \left[ {{\text{MJ}}/{\text{kg}}} \right] \, = {33}.{\text{8C }} + { 14}.{4}\left( {{\text{H}}_{{2}} - {\text{O}}_{{2}} /{8}} \right)$$

4 $${\text{L}}.{\text{H}}.{\text{V}}. \, \left[ {{\text{MJ}}/{\text{kg}}} \right] \, = {\text{H}}.{\text{C}}.{\text{V}}. - \, \left( {{\text{9H2}}*{24}.{42}} \right)$$

It is also noticed that the lower calorific value for 3a1 is lower than the 3b1 microemulsion fuel due to the increasing percentage of hydrogen formed in 3a1 microemulsion fuel. The higher the percentage of hydrogen, the higher the water formed that loses part of its calorific value due to combustion.

From these theoretical analyses of combustion, it was found that although there is a part lost in the calorific value, the use of ionic liquids saved the air consumption required for combustion, as well as reducing the quantities of combustion products.

$${\text{Weight of products of combustion }}\left[ {{\text{kg}}} \right] \, = \, \left( {{11}/{3}} \right){\text{ C}} + {\text{9H}}_{{2}}$$

it was found that microemulsion fuels need air to fuel ratio lower than that is needed for diesel. In addition, 3b1 microemulsion fuel needs air to fuel ratio lower than 3a1 microemulsion fuel because it contained an amount of oxygen in its chemical composition higher than 3a1 microemulsion fuel. The heat released from 3b1 is higher than that from 3a1 microemulsion fuel and diesel because 3b1 contain higher amounts of carbon and hydrogen.

## Conclusion

- New diammonium Ionic liquids were synthesized to enhance the solubilizing of ethanol and the stability of microemulsion samples.
- All the prepared microemulsion samples have particle sizes of ethanol between 4.77 to 11.22 nm with a very narrow size distribution as confirmed by DLS analyses.
- For 160 days, the prepared samples showed a significant stability relative to their particle sizes and no phase separation was observed for a year.
- The diquaternary nitrogen, ethylene oxide spacer and several long hydrocarbon chains help in the emulsification process by making the formed reverse micelles to be more stable and preventing phase separation.
- The microemulsions 3a1 and 3b1 samples need an air/fuel ratio lower than that is needed for pure diesel. They need 13.07 kg air/kg fuel and 12.79 kg air/kg fuel, respectively. Moreover, the prepared samples reduce the quantities of combustion products due to their improved combustion.
- The microemulsion fuel samples have slightly lower cetane indices, heating values, slightly higher kinematic viscosities and equivalent densities than the neat diesel. Nevertheless, the fuel properties fall within the ASTM D975 standards and are close to the neat diesel properties.
- The prepared microemulsions were proven to be an effective replacement for diesel since they improved the combustion properties of diesel and produced less pollution. In addition, the method of preparation is simple, and cheap and the mixing process is performed at room temperature.

## Data Availability

The datasets used and/or analyzed during the current study available from the corresponding author on reasonable request.

## Abbreviations

ILs: Ionic liquids
WCO: Waste cooking oil
*P*: Predict the particle size, (nm)
*X*: Time, (days)
*LHV*: Lower heating value, (MJ/kgf)
*HHV*: Higher heating value, (MJ/kgf)
a: Microemulsion samples contain ionic liquid (3a)
b: Microemulsion samples contain ionic liquid (3b)
DLS: Dynamic light scattering
2a: Diethyl ethanolamine tridecantrioate
2b: Triethyl amine tridecantrioate
NMR: Nuclear magnetic resonance
